# Passive eDNA Sampling Characterizes Fish Community Assembly in the Lancang River of Yunnan, China

**DOI:** 10.3390/biology14081080

**Published:** 2025-08-19

**Authors:** Li Ding, Xinbin Duan, Mingdian Liu, Daqing Chen, Xiaofeng Huang, Dengqiang Wang, Baoshan Ma, Shijian Fu, Liqiao Zhong

**Affiliations:** 1 Laboratory of Evolutionary Physiology and Behavior, Chongqing Key Laboratory of Conservation and Utilization of Freshwater Fishes, Chongqing Normal University, Chongqing 401331, China; dl918666@126.com; 2 National Agricultural Science Observing and Experimental Station of Chongqing, Yangtze River Fisheries Research Institute, Chinese Academy of Fishery Science, Wuhan 430223, China; duan@yfi.ac.cn (X.D.); lmd@yfi.ac.cn (M.L.); chdq@yfi.ac.cn (D.C.); wdq@yfi.ac.cn (D.W.); baoshanma@yfi.ac.cn (B.M.); 3 College of Animal Science and Technology, Yangtze University, Jingzhou 434025, China; xfhuang2020@163.com

**Keywords:** environmental DNA, the Lancang River, freshwater fish, passive eDNA samplers

## Abstract

This study aimed to determine the practical efficacy of passive eDNA samplers (PEDS) for monitoring fish diversity in riverine ecosystems. It investigated the utility of environmental DNA (eDNA) in accurately depicting fish composition and diversity within the Lancang River. Environmental DNA technology, particularly PEDS, may be used as a substitute for traditional water filtration techniques. However, its effectiveness in natural water ecosystems remains to be proven. The filter materials included mixed cellulose acetate and nitrate (MCE), nylon (NL), glass fiber (GF), and polyvinyl chloride filter membrane (PVC). This study used four different types of filters, each with identical pore sizes and dimensions but constructed from various materials, to assess eDNA capture under laboratory and field conditions in the water samples. The filter materials included mixed cellulose acetate and nitrate (MCE), nylon (NL), glass fiber (GF), and polyvinyl chloride filter membrane (PVC). Environmental DNA macrobarcoding was used to analyze fish biodiversity and to understand the environmental effects on species distribution. Our study identified 50 fish species inhabiting the Lancang River, with equal representation of exotic and native species. A comparative analysis of four filter-based environmental DNA samplers and traditional environmental DNA sampling methods demonstrated comparable species richness. Redundancy analysis indicated that environmental variables, elevation, electrical conductivity, salinity, and chlorophyll-a significantly influenced the distribution patterns of both non-native and native fish species in the river. This study highlights the significance of eDNA technology in evaluating fish diversity across diverse habitats, thereby establishing a theoretical framework for the sustained monitoring and management of fish biodiversity in protected areas.

## Simple Summary

The assessment of species composition within river fish ecosystems has relied heavily on fishing surveys, which are both time-intensive and labor-intensive. Environmental DNA (eDNA) technology has significantly advanced the field of biological monitoring using passive environmental DNA samplers (PEDS) instead of traditional active water filtration methods. In this study, four different types of filters were used to evaluate their proficiency in capturing eDNA from water samples that ranged from homogeneous to heterogeneous. All four filters effectively captured eDNA, with consistent detection of fish species in aquatic environments. Our study determined that the distribution patterns of both non-native and native fish species in the Lancang River were predominantly shaped by variables such as elevation, electrical conductivity, salinity, and chlorophyll-a. The application of eDNA technology for the rapid evaluation of fish diversity in extensive river ecosystems has crucial implications for the sustained monitoring and management of biodiversity in protected regions.

## 1. Introduction

Environmental DNA (eDNA) is genetic material released by organisms as they interact with their surroundings. They can be identified through sequence analysis, making them invaluable assets for comprehensive ecological research [1,2]. Water is primarily used as a medium for collecting eDNA samples [3]. Environmental DNA technology has become an important complement to traditional biological monitoring techniques, with wide-ranging applications in aquatic ecology [4,5]. This strategy aids in identifying both endangered and invasive species and exploring aquatic biodiversity [6]. Temporal dynamics significantly affect biodiversity composition [7]. For instance, whereas short-term biomass assessments can be completed within a week, understanding the seasonal succession of species necessitates approximately three weeks. In contrast, evaluating long-term species trends requires regular monthly observations. Consequently, regular sampling is essential for effectively tracking ecosystem changes. Additionally, studies have examined species distribution in vast river systems [8], whereas other studies have focused on the presence of rare species in remote and hard-to-access mountain streams [9].

Environmental DNA (eDNA) sampling has emerged as a crucial method for evaluating aquatic biodiversity in diverse environments. To further the advancement of eDNA technology, researchers have developed various innovative devices for water sampling, filtration, and eDNA preservation, including remote access samplers (RAS) [10], backpack OSMOS eDNA samplers [11], and unmanned aerial vehicle sampling technology [12]. Furthermore, underwater automated eDNA samplers, priced at approximately $280 [13], and field instruments for the automated archiving of eDNA samples in seawater have been developed [14]. Additionally, innovations such as in situ microbial filtration and immobilization [15], hydraulic suction large-scale bio-holding samplers [16], and the application of silica gel beads for in situ membrane preservation methods have been introduced [17]. In recent years, passive eDNA samplers (PEDS) have gained recognition as alternative methods for collecting eDNA samples, such as those obtained from filtered water. Research indicates that passive environmental DNA samplers (PEDS) offer significant benefits over active sampling techniques. Specifically, in environments characterized by low biomass or very low concentrations of environmental DNA (eDNA), active filtration necessitates the collection of substantial water volumes (5–10 L), which poses challenges for on-site pumping of water. Conversely, passive samplers are capable of accumulating eDNA directly from the environment over extended durations (7–30 days), thereby enhancing detection rates by a factor of two to four [18]. Active filtration systems are prone to clogging in aquatic environments with high turbidity or eutrophic conditions. Conversely, passive samplers permit the diffusion of water molecules and DNA, which significantly lowers the likelihood of clogging and results in a 70% decrease in maintenance expenses [19]. In regions characterized by remoteness or limited human resources, the deployment of a passive sampler on a single occasion is sufficient to facilitate continuous sample collection. This approach significantly reduces the necessity for multiple sampling excursions, thereby conserving both manpower and fuel [20]. Passive samplers are advantageous for evaluating alterations in diel vertical migration because they can function continuously at a fixed location for extended periods, thereby yielding time-weighted average measurements. In contrast, active sampling is limited to capturing only transient data points [21]. These PEDS can be either naturally occurring or human-made and enable the acquisition of eDNA without the need to pump water through the filter membranes. This approach eliminates the need for pumps and their associated power equipment, thereby significantly lowering the costs associated with sampling [22]. The featuring compartments filled with sorbents, such as montmorillonite clay and granular activated carbon, have been demonstrated to be highly effective for capturing eDNA in both laboratory and field settings [23]. This filter is directly submerged in the water column to collect eDNA [24], and it also provides the advantage of facilitating extensive sampling. The custom gauze rolls affixed to the interior of commercial trawls are used to capture eDNA [25]. Chen et al. [26] have illustrated that glass fiber membranes serve as an efficient and globally advantageous method for the passive collection of eDNA in aquatic settings. Given the inherent complexity and variability of these environments, along with numerous external factors influencing eDNA capture and identification, significant challenges to standardization remain in this field. These challenges include the development of uniform sampling equipment, formulation of standardized sampling protocols, and establishment of consistent methodologies for DNA extraction, amplification, and sequencing [27]. The quality of water is a critical determinant in the success of eDNA protocols [28]. Various aquatic environments, such as stagnant, flowing, surface, and deep waters, demand specific sampling equipment for accurate monitoring. Additionally, elements such as turbidity, temperature, biomass, salinity, and ultraviolet light in these water bodies are essential for the effective enrichment and detection of eDNA [29,30]. For instance, while a higher concentration of suspended particles in water correlates with increased eDNA yield, it simultaneously diminishes the efficiency of polymerase chain reaction (PCR) [31,32].

The Lancang-Mekong River, the largest in Southeast Asia and the 11th longest globally, is renowned for its remarkable aquatic biodiversity and rich fishery resources [33]. In recent years, freshwater ecosystems across the globe have witnessed a notable decrease in biodiversity [34]. Local environmental and spatial variables play pivotal roles in determining the composition and structure of fish communities [35,36]. The Lancang River flows through regions marked by towering mountains and profound gorges, characterized by substantial elevation changes and rapid current. Implementing traditional net fishing in these areas poses significant challenges and may inadvertently lead to the capture of rare fish species, potentially underestimating their biodiversity. Between 2008 and 2015, researchers used traditional fishing techniques to collect data on fish species residing in the Lancang River. The study identified 162 native fish species in the river. However, over the past three decades, 49 of these native species have vanished from the river’s middle and lower reaches, whereas 22 non-native species have successfully established themselves. The combined effects of cascade dams and invasive species in the downstream main channel have led to an average loss of nearly half of the native species in each section [37]. Environmental DNA (eDNA) analysis, which relies solely on water samples, is a highly sensitive and noninvasive method for detecting species that are either elusive or low in abundance. This technique is rapid, cost-efficient, and equally effective in fast-moving and deep waters, making it an optimal approach for investigating complex aquatic ecosystems [38]. eDNA can be used to simultaneously identify all species present within a 10 km vicinity of a water body. It is also effective in detecting species that exist in low-density populations and are rare. By analyzing the relative abundance of these species, researchers can determine whether the organisms exhibit high metabolic activity. Environmental DNA (eDNA) technology has emerged as a crucial tool for conserving biodiversity, especially as environmental stressors continue to increase [39,40,41]. Environmental DNA (eDNA) analysis has some challenges. One main issue is that DNA can break down and move in water. This makes it difficult to determine the actual distribution of species [42]. Environmental DNA is limited in its ability to precisely measure species population size, individual body dimensions, age structure, sex ratio, and reproductive status. Furthermore, environmental DNA analysis requires a sequencing platform [43].

This study aimed to evaluate fish composition and determine the environmental factors influencing fish distribution in the Lancang River. This study employed a passive eDNA sampling method using four cost-effective and standardized filter membranes, with two main objectives: (1) elucidate the effects of passive eDNA sampling across various environments and (2) identify the factors influencing changes in the diversity of native and non-native fish species in the Lancang River. In this study, we aimed to explore the feasibility of using PEDS techniques in the Lancang River. The results support regional authorities and researchers in crafting cost-effective and efficient biosafety monitoring strategies as well as in assessing the impact on fish distribution patterns in the region.

## 2. Materials and Methods

Four different membrane materials, mixed cellulose acetate and nitrate (MCE), nylon (NL), glass fiber (GF), and polyvinyl chloride filter membrane (PVC) (all with a standard 50 mm diameter and 0.45 μm pore size), were used in the passive eDNA samplers (Hangzhou Special Paper Industry Co., Ltd., Hangzhou, China). Contamination was minimized by wearing gloves and using sterile tweezers to handle each filter membrane. The membrane materials were stored individually in sterile 10 mL centrifuge tubes at −20 °C until eDNA extraction. Each experiment was performed in triplicate. Environmental DNA (eDNA) extraction was performed using the TIANamp Stool DNA Kit (Tiangen Biotech, Beijing, China) according to the manufacturer’s instructions, and the final DNA extract was reconstituted in 50 µL of transformation buffer. The concentration of eDNA was quantified using a NanoDrop 2000 spectrophotometer (Thermo Fisher Scientific, Waltham, MA, USA).

### 2.1. Effect of Time and Density on eDNA Absorption

Laboratory experiments were conducted in rectangular water tanks (38 cm × 26 cm × 24 cm) at 24 °C, which offered a relatively controlled system containing known fish species (body length 5.3–6.1 cm, weight 1.2–1.5 g). The experimental tanks and one negative control tank were set up in a room that had never been exposed to fish before. All tanks were treated with a 10% bleach solution overnight, subsequently washed with distilled water, and subjected to a final wash with sterile deionized distilled water. Each tank was filled with 20 L of tap water, which was allowed to sit for three days to dechlorinate. The fish were introduced into the experimental tank and allowed to acclimatize for 3 days before the experiment.

**Figure 1:**
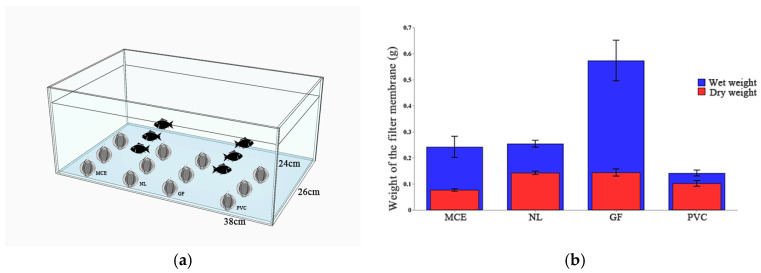
Passive eDNA collection experimental apparatus, design, and performance: (**a**) experimental apparatus containing filter membranes encased in hollow metal spheres that were submerged in water; (**b**) experimental design showing dry and wet weights (30 min soak) of four filters (GF, PVC, MCE, and NL).

An experiment was conducted to investigate the effect of submersion time on the concentration of captured eDNA in the four membrane types. To start the experiment, four membrane materials were placed into hollow perforated metal spheres with a 50 mm diameter and submerged at the bottom of the water (Figure 1a).

Three replicates were retrieved from each tank at each of the four submersion time points, 15, 30, 60, and 120 min. To investigate the effect of biomass density on the concentration of captured eDNA using the four membrane materials, three replicates were retrieved from each tank at each of the seven biomass densities: 0, 0.5, 1.0, 1.5, 2.0, 2.5, and 3.0 tails/L. Furthermore, we used the gravimetric method to quantitatively investigate the water absorption properties of the filter membranes for each of the different materials.

### 2.2. Fish Biodiversity Detection Capacity

**Figure 2:**
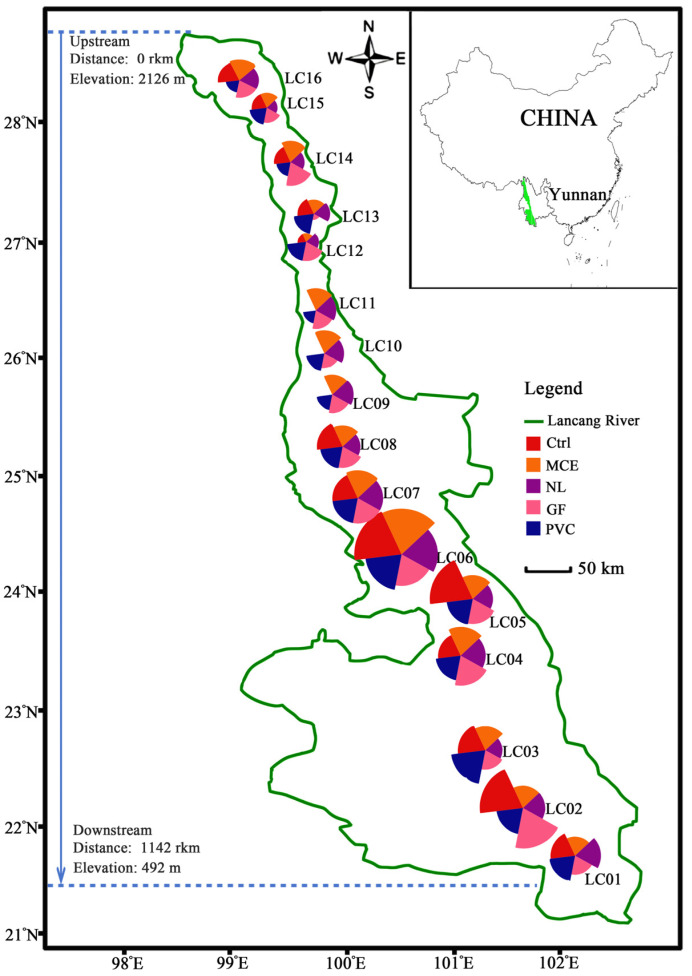
The 16 sampling sites in the river. The four membrane materials were compared to the number of traditionally filtered eDNA samples (labeled Ctrl). Four membrane materials were used in the passive eDNA samplers—MCE: mixed cellulose acetate and nitrate, NL: nylon, GF: glass fiber, and PVC: polyvinyl chloride filter membrane (all with a standard 50 mm diameter and 0.45 μm pore size). Each sector is uniquely colored to signify either a particular filter membrane or a traditional filtration method. The dimensions of each sector reflect the number of fish identified.

A field experiment was performed on the Lancang River in Yunnan, China. Passive eDNA samplers (PEDS) were conducted between 8 a.m. and 4 p.m. on 11–15 August 2024 at 16 sites (LC01–LC16) around the river (Table S1 in the Supplementary Materials) by submerging four different membrane materials just below the metal-perforated ball with a tether (Figure 1a). The locations of the sampling sites are shown in Figure 2. These sites spanned a total shoreline distance of approximately 1142 km and covered the biodiverse Lancang River area in Yunnan Province.

The fish biodiversity detected by the four membranes was examined after 30 min to assess eDNA collection. We deployed four different membrane materials in the river, and three replicate filters were retrieved from each site. For comparison, 15 L river water samples (labeled as control) were collected from the shore at each site prior to installation of the PEDS, and each sample was vacuum-filtered through a filter from Easy Filter Kit User Guide (Nanjing E-genomics Technology Co., Ltd., Nanjing, China) on the same day.

### 2.3. Measurements of Environmental Variables

A total of twelve environmental factors were involved in this study (Figure S1, for detailed information, in the Supplementary Materials) and analyzed by a portable multiparameter meter YSI ProQuatro (YSI Incorporated, Yellow Springs, OH, USA) and Turner Designs AquaFluor (Turner Designs, San Jose, CA, USA): latitude (Lat), longitude (Long), atmospheric pressure (AP), elevation (Elev), electrical conductivity (EC), water temperature (WT), dissolved oxygen (DO), total dissolved solids (TDS), salinity (SAL), pH, turbidity (Turb), and chlorophyll-a (Chl.a).

### 2.4. Biodiversity Assessment

The PCR primer sequences of 12S rDNA were Tele02-F (5′-AAACTCGTGCCAGCCACC-3′) and Tele02-R (5′-GGGTATCTAATCCCAGTTTG-3′). A library was constructed with the DNA Library Prep Kit and sequenced by a PE300 platform (Biozeron Co., Ltd., Shanghai, China). The raw reads were quality-controlled and filtered with Fastp (v0.12.4). Paired-end reads were merged and filtered with USEARCH (v11.0.667). Replicated and low-abundance sequences were removed by VSEARCH (v2.15.2). Chimeras were removed, and sequences with similarity > 98% were clustered as operational taxonomic units (OTUs). The obtained OTUs were blasted with the MitoFish database (v3.85). Only fish species with identities ≥ 90% and sequence variants that could be assigned to at least the species level were included. All fish species were divided into native and non-native fish species by referring to the species inventories provided in previous studies [44].

### 2.5. Statistics

One-way analysis of variance (ANOVA) and subsequent Tukey’s HSD post hoc tests were used to compare eDNA yield across adsorbent materials and fish species richness detected in the field experiment. Fish species diversity within communities was assessed using a range of diversity indices, specifically the Chao 1, Shannon, and Simpson indices. To evaluate the fish communities identified through various filter membranes, principal coordinate analysis (PCoA) was applied, utilizing the Bray–Curtis similarity distance matrix to determine community similarity. Differences in community structure across different filter membrane treatment groups were analyzed using permutational multivariate analysis of variance (PERMANOVA). Furthermore, redundancy analysis (RDA) was performed to investigate the influence of environmental variables on the structure of fish communities. The threshold for statistical significance was set at *p* < 0.05. All statistical analyses and plotting were performed in R version 4.1.0 (R Development Core Team 2021; https://www.r-project.org/ (accessed on 5 August 2025).

## 3. Results

### 3.1. Materials Enable Passive eDNA Collection

**Figure 3:**
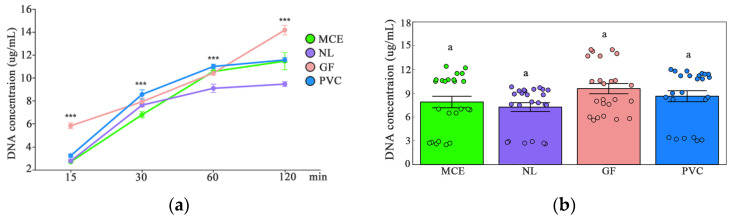

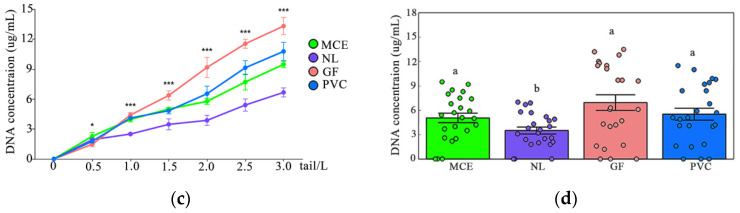
eDNA of samples obtained by laboratory PEDS method: (**a**) different soaking time, eDNA enrichment, *** indicates significance at the 0.1% level (*p* < 0.001); (**b**) eDNA enrichment at different times, and different letters indicate statistical significance (a = 0.05); (**c**) different biomass, eDNA enrichment, * indicates significance at the 5% level (*p* < 0.05), and *** at the 0.1% level (*p* < 0.001); (**d**) biomass eDNA enrichment, and different letters indicate statistical significance (a = 0.05).

The four filter membranes (MCE, NL, GF, and PVC) used in the laboratory experiment were selected for evaluation to determine the suitable submersion time and to conduct performance testing of each membrane. All four membranes collected detectable eDNA. The DNA concentration of all four filter membranes increased with increasing submersion time; however, significant differences in the mean concentration values were observed between the membranes (Figure 3a). GF was the only filter membrane that showed a strong increase in eDNA yield with a rise in the four membranes. Additionally, no significant differences in mean concentration values were observed at the four stages of time (Figure 3b). Given the constraints of field sampling, the submersion time for field sampling was set to 30 min.

The DNA concentration of the four filter membranes increased with an increase in bio-density, ranked as GF, PVC, MCE, and NL (Figure 3c), but significant differences in mean concentration values existed at NL, with a downward trend (Figure 3d). Among these, the filter membrane GF exhibited the greatest weight change after water absorption at 75% (Figure 1b).

### 3.2. The Majority of Fish Species Were Detected with All Materials

**Figure 4:**
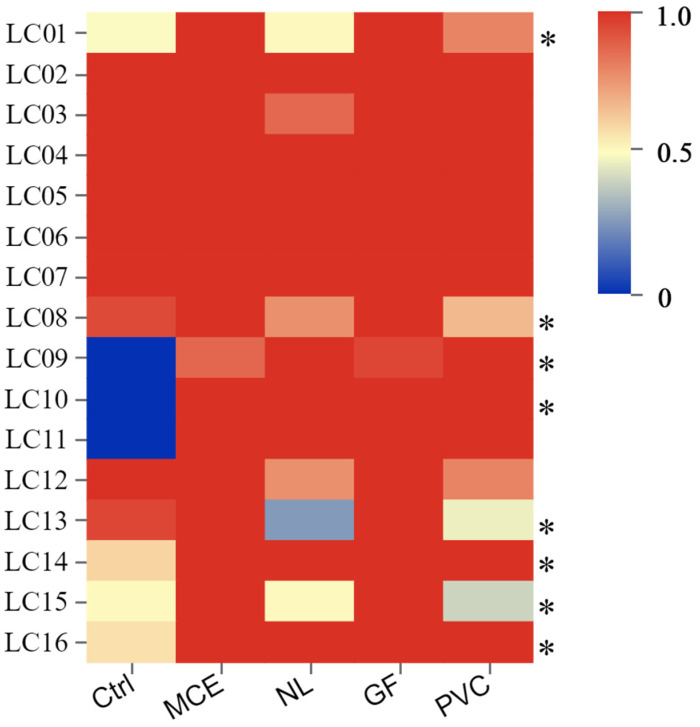
Heatmap showing the eDNA concentrations (normalized) detected in the Lancang River using four filters as passive eDNA samplers in Lancang River. Filters were deployed for 30 min at 16 locations along the Lancang River, with three replicates at each location. In addition, the results of enrichment of eDNA by filtering three replicates of 5 L water samples at each site are also shown (Ctrl). The darker red blocks represent higher DNA concentration, while blue blocks indicate lower concentration. It represents active filtration (labeled: Ctrl). The sample is filtered after standing because of turbidity (turbidity > 30 NTU). The unmarked * is the filtered water sample on the spot.

The membranes tested differ in their ability to adsorb eDNA when used as passive sampling devices (PEDS). The eDNA levels of all adsorbed materials from the 16 field sites were detectable by concentration after eDNA extraction, whereas the control did not extract DNA at three sites, Laomi mountain (LC09), Yingpan town(LC10), and Shideng township (LC11) (traditional water filtration to adsorption of eDNA), but obtained it in passive sampling equipment. GF and MCE showed stable eDNA yields among the four membranes (Figure 4).

**Figure 5:**
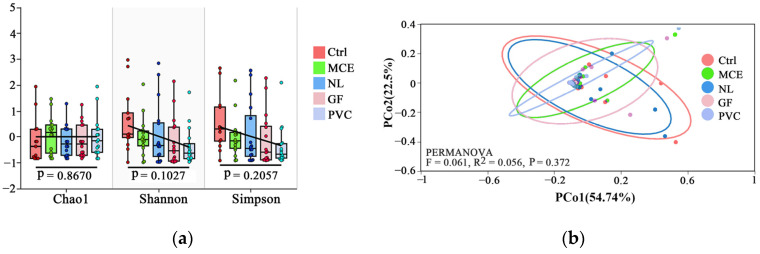
Comparisons of fish species richness detected by eDNA metabarcoding of samples acquired using water filtration (labeled: Ctrl) and PEDS: (**a**) alpha diversity (Chao1, Shannon, and Simpson index) based on number of species detected at 16 sampling points; (**b**) principle coordinate analysis (PCoA) plots of fish assemblages recovered using water filtration and PEDS (four filter membrane materials) at 16 sites.

For all materials, we detected 9,590,800 sequence reads, with an average length of 13,650, and 5,811,075 OTU (operational taxonomic unit) reads (Figure S2, for detailed information, in the Supplementary Materials.). The α-diversity detected did not differ between materials, indicating that the diversity detected in all materials was comparable to that of the conventionally filtered eDNA samples (Figure 5a; *p* > 0.01), whereas MCE and PVC detected the lowest fish diversity on average.

To further assess the spatial effects of the two sampling methods, we compared the spatial variation in eDNA–detected fish assemblages detected by water filtration and PEDS sampling using PCoA (principal coordinate analysis). A slight difference was found between the qualitative assemblage compositions detected by the two methods across sampling sites, but no significant quantitative difference was found (PERMANOVA, R^2^ = 0.0561, *p* > 0.01; Figure 5b).

**Figure 6:**
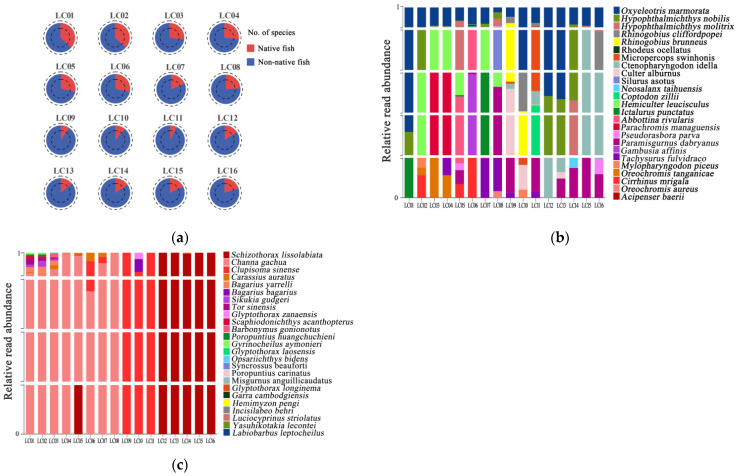
Composition of fish communities at different sample sites by analyzing the relative abundance of reads of different taxa at the species level: (**a**) the composition of fish communities at different sampling sites, including non-native and native species; (**b**) composition of non-native fish; (**c**) composition of native fish.

A total of 50 fish species were detected in the Lancang River. The number of fish species detected varied between filter membranes (Figure 2), and all membranes detected species in numbers comparable to conventionally filtered eDNA samples. The range of fish species detected by material was 14 (filtered eDNA; range = 0–36), 15 (MCE; 4–29), 13 (NL; 5–23), 14 (GF; 3–29), and 15 (PVC; 6–30). An increase in elevation was associated with a notable reduction in fish species diversity. Conversely, at lower elevations, exemplified by site LC06 (Xiyi Wharf), there was a pronounced increase in the number of species documented (Figure 6a). The 50 species are evenly divided between native and non-native species, each constituting 50% of the total. The proportions of non-native and native fish species at each site are shown in Figure 6a. The proportion of non-native species ranged from 63.1% to 92.5%, with the largest site being LC11 (92.5%); the proportion of native species ranged from 7.5% to 36.9%, with the first three lowest being LC9 (9.4%), LC10 (10.9%), and LC11 (7.5%). Environmental DNA sampling was carried out at 16 sites to assess fish community composition, as reflected by relative read abundance. Native fish, such as *Schizothorax lissolabiata*, were predominantly found in river samples LC12–LC16, while *Channa gachua* was primarily observed in river samples LC01–LC08 (Figure 6b). Non-native fish exhibited relatively high relative abundance, with notable species including *Oxyeleotris marmorata*, *Hypophthalmichthys nobilis*, and *Hypophthalmichthys molitrix* (Figure 6c).

### 3.3. Relationships Between Environmental Factors and Community Structures

**Figure 7:**
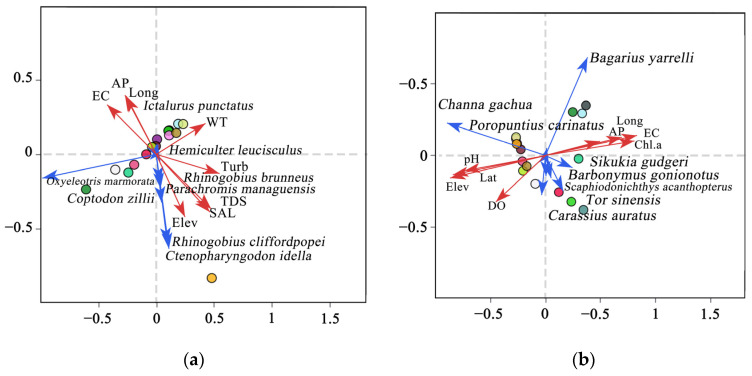
The RDA ordination graphic depicts the relationship between fish community structure and the environmental factors that were measured: (**a**) the relationship between non-native fish and environmental factors; (**b**) the relationship between native fish and environmental factors. The red arrows, blue arrows, and colored circles indicate the following—red vectors: environmental variables correlated with fish community structure; blue vectors: these denote fish community structures; colored circles indicate the distribution of each sampling site. The vector length indicates the strength of the influence, whereas the angle signifies the correlation (an acute angle indicates a positive correlation).

Redundancy analysis (RDA) was employed to investigate the correlation between fish communities and environmental variables (Figure 7). The analysis revealed results pertaining to non-native species and environmental factors, as depicted in Figure 7a. Specifically, the findings indicated a positive association between the distribution of *Oxyeleotris marmorata* and electrical conductivity (EC), atmospheric pressure (AP), and longitude (Long), while the distribution of *Rhinogobius cliffordpopei*, *Ctenopharyngodon idella*, *Coptodon zillii*, *Parachromis managuensis*, and *Hemiculter leucisculus* exhibited positive correlations with elevation (Elev), salinity (SAL), total dissolved solids (TDS), and turbidity (Turb). Conversely, *Oxyeleotris marmorata* showed a negative correlation with these variables. The results presented in Figure 7b illustrate the relationship between native fish species and environmental variables. *Bagarius yarrelli*, *Poropuntius carinatus*, and *Sikukia gudgeri* distribution exhibited positive correlations with chlorophyll-a (Chl.a), electrical conductivity (EC), atmospheric pressure (AP), and longitude (Long). Conversely, *Channa gachua* distribution was positively associated with pH, elevation (Elev), latitude (Lat), and dissolved oxygen (DO). *Tor sinensis* and *Barbonium gonionotus* distributions were positively correlated with dissolved oxygen (DO).

## 4. Discussion

### 4.1. Evaluation of the Ability of Four Materials to Passively Collect Environmental DNA in Laboratory Environments

Passive samplers (PEDS) play a significant role in environmental DNA (eDNA) technology. We assessed the efficacy of four cost-effective materials for the passive collection of environmental DNA (eDNA) within a 20 L small-scale experimental system. This evaluation was conducted by measuring the immersion time, biological density, and weight of the filter after absorption. We conducted an experiment by immersing materials with consistent pore size and diameter (MCE, NL, GF, and PVC) in water and discovered that these four economically viable materials were notably effective in the passive collection of environmental DNA (eDNA) from water. In this study, the eDNA traditional filtration system necessitated the use of manufacturer-specific disposable glass fiber HEPA filter membranes (Nanjing E-genomics Technology Co., Ltd., Nanjing, China), each priced at approximately 200 yuan. Conversely, laboratory-grade universal filter membranes, such as MCE, NL, and GF, are available for approximately 0.5 yuan each, and PVC membranes cost around 2 yuan, all intended for single use. This indicates that the traditional specialized membrane is 400 times more expensive than the MCE/NL/GF membranes and 100 times more costly than the PVC membranes, highlighting a significant cost disparity. Our analysis of the soaking duration indicated that the capture efficiency of each material improved over time; however, no statistically significant variation was observed in the average capture efficiency with respect to the soaking duration. According to Bessey et al., fish species richness determined through enriched eDNA after a 5 min immersion was comparable to that obtained using traditional filtration techniques for most filter materials [45]. Among the materials tested, GF consistently yielded the highest eDNA capture with prolonged soaking times (15–120 min), whereas NL exhibited the lowest average yield, followed by MCE and PVC. Additionally, our investigation into biological density revealed that the capture efficiency of each material increased with higher biological density, and there were notable differences in eDNA collection efficiency across different materials. GF and PVC demonstrated superior performance compared with MCE and NL. In terms of biological density, GF emerged as the most effective material, collecting the highest average eDNA, whereas NL was the least effective. GF is capable of passively capturing aquatic eDNA, with efficiency comparable to or exceeding that of traditional methods. Upon comparing the weights of the four materials post-water absorption, it was observed that GF exhibited the greatest weight change, whereas PVC exhibited the least. The selection of the material is influenced by factors such as cost, robustness, ease of deployment, and ease of subsequent processing. The efficacy of GF in capturing eDNA is likely attributable to its substantial water absorption capacity. Chen et al. noted that GF did not reach saturation even after three days of immersion and continued to adsorb eDNA [26]. However, the volumetric expansion of GF upon water absorption increases the workload associated with eDNA extraction in subsequent processes. Previous studies have indicated that MCE is the most efficient medium for capturing eukaryotic environmental DNA (eDNA) in clean water settings, although it is prone to clogging. NL is noted for its resistance to organic solvents and ability to withstand repeated washing [20]. GF is highly sensitive to free DNA; however, its fibers are susceptible to shedding [46]. PVC mesh is an economical option that can be washed multiple times, making it ideal for processing large volumes of turbid water; however, it is less effective at capturing DNA from larger particles [47].

To enhance sampling capacity, future research is likely to increasingly employ passive eDNA collection techniques in complex aquatic environments. However, these methods will be influenced by factors such as water flow and quality. The optimization of eDNA sampling in turbid water has been crucial in determining the concentration of extracted eDNA [48]. Environmental DNA (eDNA) manifests in various forms, sizes, and molecular states within natural ecosystems, and the chemical composition of water at different sites can influence the efficacy of passive eDNA collection methods [49,50]. To further assess their efficacy in passive eDNA collection, we deployed these four materials in the Lancang River, located in Yunnan, a region renowned for its high species diversity and complex hydrological conditions.

### 4.2. Evaluation of the Ability of Four Materials to Passively Collect Environmental DNA in the Lancang River

In laboratory ecosystems, no significant differences in mean capture efficiency were detected among the materials at varying immersion times. Considering the high mobility and low biomass density of the aquatic ecosystem of the Lancang River, extending the sampling duration could improve the detection of rare species and underscore the limitations of passive eDNA sampling methods in capturing water volume data. We opted for a 30 min immersion period for passive eDNA sampling in the Lancang River, alongside the conventional filtration of 15 L of water as a control measure. Our study revealed that the species count and taxonomic diversity identified using environmental DNA (eDNA) captured by PEDS were comparable to those detected using conventional water filtration methods. Notably, eDNA was absent in the traditionally filtered water samples at three sites (LC09, LC10, and LC11). At sites LC09 and LC10, high turbidity levels (exceeding 30 NTU) necessitated filtering of the water samples after a period of settling, which resulted in no eDNA capture. Similarly, at site LC11, on-site filtration failed to capture eDNA. The underlying causes of eDNA degradation at these sites remain unclear and require further investigation to be elucidated. Despite this, our research demonstrates that eDNA was successfully captured by all four materials tested, indicating that PEDS can effectively mitigate the challenges of eDNA degradation encountered with traditional filtration methods. The results of the principal coordinates analysis (PCoA) indicated a partial overlap in the species numbers captured by PEDS and conventional filtration, corroborating the findings of previous research [51]. Furthermore, the capture of eDNA by PEDS in natural settings may be influenced by biological factors, such as the stochastic nature of species emergence and the random processes of eDNA release, diffusion, and capture. Additionally, abiotic factors, including pH, oxygen content, conductivity, temperature, and salinity, may play a role. Consequently, to enhance the efficiency and accuracy of species detection using eDNA across various habitats, improvements and further efforts in field sampling design should be undertaken [52,53].

This study revealed that, at the Xiyi Wharf (LC06) sampling location, there was a direct relationship between species diversity and the number of fish identified. The expansion of rural tourism activities in the vicinity of Xiyi Wharf, which offers tourists opportunities to participate in fishing and aquaculture, seems to contribute to the elevated fish counts observed in this region. This study robustly supports the application of environmental DNA (eDNA) as a tool for evaluating fish community structures across various habitats, a finding corroborated by previous research [54,55]. Our analysis indicated that 50 distinct fish species were recorded in the survey data from the Lancang River [44]. Approximately 50% of these species are native. Native species, such as *Schizothorax lissolabiata* and *Channa gachua*, are prevalent, while non-native species, including *Oxyeleotris marmorata*, *Hypophthalmichthys nobilis*, and *Hypophthalmichthys molitrix*, also exhibit dominance. Our findings indicate that the proportion of native species varies from 7.5% to 36.9%. The analysis of the upstream-to-downstream gradient (LC16–LC01) revealed that native fish diversity is greater in downstream areas than in upstream regions. This finding is consistent with the river continuum concept, which suggests that spatial patterns of species abundance tend to increase downstream. This increase is likely due to the enhanced availability of material and energy resources in the downstream or interaction zones [56]. In comparison to traditional fish species surveys conducted in the Lancang River, which identified 40 species through netting and 45 species via electrofishing, with an average species richness of 3.5–4.0 species per site [57], this study employed environmental DNA (eDNA) analysis to detect 50 fish species across the entire Lancang River Basin. The average species richness increased to 4.5 species per site. The use of eDNA sampling from water has successfully addressed the limitations of conventional equipment concerning water depth and microhabitats, thereby offering more extensive spatial coverage and improved detection sensitivity compared to conventional equipment.

Future research should integrate the four-dimensional index of “adsorption efficiency-filtration speed-reuse-economic cost” into a comprehensive framework to identify the most appropriate filter membrane for various research objectives. Candidate filters should be evaluated concurrently in freshwater, saltwater, and high-turbidity environments to systematically assess their ecological versatility. Although environmental DNA (eDNA) offers significant advantages in monitoring fish diversity, traditional methods, such as net fishing and electrofishing, retain indispensable complementary value in specific contexts. Therefore, the development of new filter materials with high capture rates and low costs should be encouraged to facilitate the broader application of eDNA technology.

### 4.3. Relationship Between Fish Diversity Distribution and Environmental Factors

Environmental and spatial dynamics play pivotal roles in shaping fish composition and structure. The introduction of non-native fish species adds new structural and functional dimensions to local ecosystems, prompting shifts in trophic dynamics [58]. Direct interactions, such as competition and predation, between non-native and native species and indirect alterations to habitats can further deplete native fish populations [59]. This study examined the interplay between fish communities and environmental factors in the Lancang River. The results revealed that the environmental determinants of non-native fish distribution differed from those of native fish. The distribution of non-native fish species is primarily influenced by factors such as elevation (Elev), salinity (SAL), total dissolved solids (TDS), and turbidity (Turb). Species such as *Rhinogobius cliffordpopei*, *Ctenopharyngodon idella*, *Coptodon zillii*, *Parachromis managuensis*, and *Hemiculter leucisculus* are particularly affected by these environmental parameters. Conversely, the distribution patterns of native fish species, namely *Bagarius yarrelli*, *Poropuntius carinatus*, and *Sikukia gudgeri*, show a positive correlation with chlorophyll-a (Chl. a), electrical conductivity (EC), atmospheric pressure (AP), and longitude (Long). Non-native fish are noted for their lower water quality requirements, which facilitates their rapid growth, heightened competitiveness, and enhanced ability to recover populations, allowing them to swiftly adapt to new environmental pressures [40].

The key environmental determinants of fish composition are water temperature, salinity, and dissolved oxygen levels [60]. Salinity significantly impacts various biological aspects of fish, including their growth, physiological and biochemical parameters, biomarkers, size and quantity of ionic cells, genes responsible for salinity regulation, and hormone levels [61]. Chlorophyll-a concentrations are indicative of organic matter introduced into estuaries via river runoff. This pigment is a marker of phytoplankton proliferation, where an increase in phytoplankton biomass is associated with elevated primary productivity, thus enhancing the environment for the development of fish food [62]. Environmental parameters, such as altitude, temperature, flow velocity, dissolved oxygen (DO), and pH, generally shape fish diversity [63,64]. Water temperature significantly influences fish diversity, primarily through its impact on the survival and reproductive success of fish species [65]. Water quality indicators, such as total dissolved solids (TDS) and electrical conductivity (EC), play a crucial role in determining the distribution of fish populations [66]. Additionally, research has highlighted the importance of elevation in shaping fish community diversity, with higher altitudes restricting the variety of fish species in riverine environments [67]. Consequently, the findings of this segment of this study indicate that non-native and native fish exhibit distinct ecological behaviors. These ecological behaviors are intricately linked to environmental changes, which may have significant implications for environmental management and fish conservation efforts. Given the observed alterations in fish communities resulting from varying levels of human influence, it is increasingly valuable to investigate the underlying causes of these differences. As human-induced modifications of the natural environment intensify, the influence of environmental factors on fish community structure is likely to become more pronounced in the future. Therefore, the outcomes of this study are relevant for future strategies in environmental management and conservation of fish diversity. Four materials that are both affordable and easily accessible are consistently produced, cost-effective, and suitable for deployment in challenging environments. Moreover, filter-based PEDS can be seamlessly integrated into existing eDNA analysis workflows through filtration methods without necessitating changes to eDNA extraction protocols. The ease of deploying PEDS, coupled with the significant reduction in effort by eliminating complex water handling and filtration tasks, facilitates eDNA surveys with enhanced spatiotemporal sampling intensity and increased biological repetitions. This advancement offers new possibilities for investigating research questions across broader spatiotemporal dimensions in the future. This study specifically focused on fish species in the Lancang River. Future investigations may consider the flexible selection of multiple filters in natural river settings or the integration of passive sampling methods to enhance capture efficiency.

It is crucial to investigate the presence of both non-native and native fish species in the Lancang River in a cost-effective manner and examine the environmental factors influencing their distribution. Our objective was to optimize on-site eDNA capture, minimize degradation during transport and storage, and ensure successful isolation and amplification. Future methodologies that address the current limitations of eDNA technology and enhance species detection may facilitate a more comprehensive assessment of biodiversity.

## 5. Conclusions

This study systematically assessed the efficacy of four different filter types for identifying fish species. Traditional environmental DNA (eDNA) techniques are hindered by turbidity, leading to inadequate eDNA collection, whereas passive samplers effectively gather additional samples. By employing passive samplers, we initiated a pioneering study on the relationship between fish distribution and environmental variables in the Lancang River, Yunnan. The methodological strengths of efficiency and precision present a new framework for conducting frequent, noninvasive studies on fish resources and ecological conservation in complex mountainous river environments.

## Data Availability

The raw data supporting the conclusions of this article will be made available by the corresponding author on request.

## Supplementary Materials

The following supporting information can be downloaded at: https://www.mdpi.com/article/10.3390/biology14081080/s1. Figure S1: The environmental factors at sixteen sites. Figure S2: Comparisons of fish species richness detected by eDNA metabarcoding of samples acquired using water filtration (labeled: Ctrl) and PEDS; the number of species detected, the average length of sequencing, and the number of OTUs detected are displayed at 16 sampling points; water filtration at three sampling sites (LC9–LC11) failed to extract and capture sufficient eDNA. Table S1: The names of sampling site.
