# Supplementary material for: Passive eDNA Sampling Characterizes Fish Community Assembly in the Lancang River of Yunnan, China
Source: Biology (Basel). 2025 Aug 19;14(8):1080. doi: 10.3390/biology14081080 (PMC12383293; doi:10.3390/biology14081080)
Supplement: Supplementary file 1 [file biology-14-01080-s001.zip › biology-3802737-supplementary.pdf]

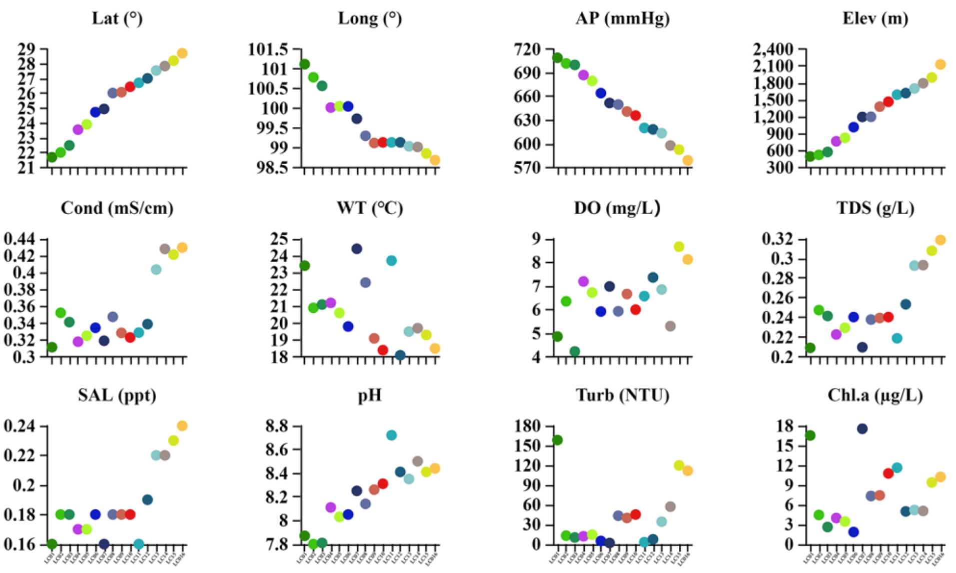


Figure S1: The environmental factors at sixteen sites.

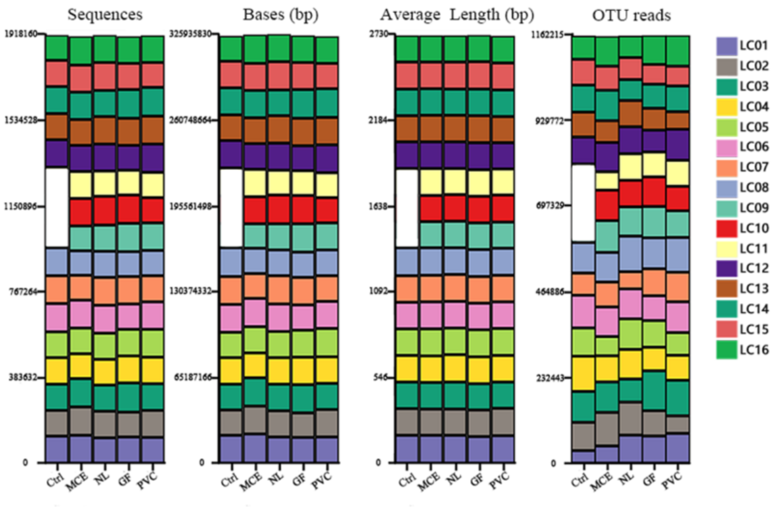


Figure S2: Comparisons of fish species richness detected by eDNA metabarcoding of samples acquired using water filtration (labeled: Ctrl) and PEDS, the number of species detected, the average length of sequencing, and the number of OTUs detected is displayed at 16 sampling points, and for traditional water filtration with three sampling sites (LC9–LC11) failed to extract and capture sufficient eDNA.

Table S1 The names of sampling site

| Sampling site | Name |
| --- | --- |
| LC01 | Guanlei Port |
| LC02 | Jing Hong |
| LC03 | Simao Port |
| LC04 | Nuozhadu |
| LC05 | Xigui warf |
| LC06 | Xiyi warf |
| LC07 | Jiangqiao warf |
| LC08 | Wayao town |
| LC09 | Laomi mountain |
| LC10 | Yingpan town |
| LC11 | Shideng township |
| LC12 | Zhonglu township |
| LC13 | Yong’an village |
| LC14 | Badi town |
| LC15 | Deigong bridge |
| LC16 | Foshan township |
